# Zebrafish Mutants Carrying Leptin a (*lepa*) Gene Deficiency Display Obesity, Anxiety, Less Aggression and Fear, and Circadian Rhythm and Color Preference Dysregulation

**DOI:** 10.3390/ijms19124038

**Published:** 2018-12-13

**Authors:** Gilbert Audira, Sreeja Sarasamma, Jung-Ren Chen, Stevhen Juniardi, Bonifasius Putera Sampurna, Sung-Tzu Liang, Yu-Heng Lai, Geng-Ming Lin, Ming-Chia Hsieh, Chung-Der Hsiao

**Affiliations:** 1 Department of Chemistry, Chung Yuan Christian University, Chung-Li 32023, Taiwan; gilbertaudira@yahoo.com (G.A.); sreejakarthik@hotmail.com (S.S.); 2 Department of Bioscience Technology, Chung Yuan Christian University, Chung-Li 32023, Taiwan; stvn.jun@gmail.com (S.J.); boni_bt123@hotmail.com (B.P.S.); stliang3@gmail.com (S.-T.L.); 3 Department of Biological Science & Technology College of Medicine, I-Shou University, Kaohsiung 82445, Taiwan; jrchen@isu.edu.tw; 4 Department of Chemistry, Chinese Culture University, Taipei 11114, Taiwan; lyh21@ulive.pccu.edu.tw; 5 Laboratory of Marine Biology and Ecology, Third Institute of Oceanography, State Oceanic Administration, Xiamen 361005, China; 6 Division of Endocrinology and Metabolism, Department of Internal Medicine, Changhua Christian Hospital, Changhua 50094, Taiwan; 7 Center of Nanotechnology, Chung Yuan Christian University, Chung-Li 32023, Taiwan; 8 Center of Biomedical Technology, Chung Yuan Christian University, Chung-Li 32023, Taiwan

**Keywords:** leptin, obesity, zebrafish, behavior, anxiety, aggression, fear, circadian rhythm, color preference, genome editing, TALEN

## Abstract

Leptin, a hormone secreted by peripheral adipose tissues, regulates the appetite in animals. Recently, evidence has shown that leptin also plays roles in behavioral response in addition to controlling appetite. In this study, we examined the potential function of leptin on non-appetite behaviors in zebrafish model. By using genome editing tool of Transcription activator-like effector nuclease (TALEN), we successfully knocked out leptin a (*lepa*) gene by deleting 4 bp within coding region to create a premature-translation stop. Morphological and appetite analysis showed the *lepa* KO fish display a phenotype with obese, good appetite and elevation of Agouti-related peptide (AgRP) and Ghrelin hormones, consistent with the canonical function of leptin in controlling food intake. By multiple behavior endpoint analyses, including novel tank, mirror biting, predator avoidance, social interaction, shoaling, circadian rhythm, and color preference assay, we found the *lepa* KO fish display an anxiogenic phenotype showing hyperactivity with rapid swimming, less freezing time, less fear to predator, loose shoaling area forming, and circadian rhythm and color preference dysregulations. Using biochemical assays, melatonin, norepinephrine, acetylcholine and serotonin levels in the brain were found to be significantly reduced in *lepa* KO fish, while the levels of dopamine, glycine and cortisol in the brain were significantly elevated. In addition, the brain ROS level was elevated, and the anti-oxidative enzyme catalase level was reduced. Taken together, by performing loss-of-function multiple behavior endpoint testing and biochemical analysis, we provide strong evidence for a critical role of *lepa* gene in modulating anxiety, aggression, fear, and circadian rhythm behaviors in zebrafish for the first time.

## 1. Introduction

### 1.1. Leptin Plays a Canonical Role on Appetite Control

Leptin, a blood-transported hormone secreted by adipocytes, circulates through blood–brain barrier (BBB) into the brain and binds to its receptor (LepR), which has been known to play a negative role in appetite control [1]. Leptin is encoded by *obese* (*ob*) gene, which is highly conserved and shares extensive homology between species [2]. The serum level of leptin is correlated with the mass of adipose tissue and increases when obese [3]. However, leptin resistance results in failure to control weight, with high level of leptin expression in the plasma, and leads to obesity [4]. On the other hand, leptin or LepR deficiency increases appetites, energy uptake, and leads to obesity in animal models and humans [5,6]. Synthetic leptin protein injection significantly reduces body weight and has been used as treatment for overweight symptom in the clinical setting [7].

### 1.2. Leptin’s Non-Appetite Controlling Characteristic

In addition to the canonical appetite control, previous studies show the effects of leptin go beyond dietary. In rodent models, leptin pathway is found to associate with myriad physiological processes other than dietary control, including body temperature, energy storage, immune response, reproduction and development [1]. Leptin-mediated immunological function is reported to promote T cell differentiation and proliferation [8,9], suggesting that leptin shares structural and functional similarities with cytokines [10,11], and may regulate innate immune responses [12]. Furthermore, evidence demonstrates that leptin regulates pubertal transition in reproduction [13,14]. More recently, leptin has drawn attention with its influences on bone metabolism through activating fibroblast factor 23 (FGF-23) and osteocalcin [15,16]. Interestingly, while leptin is thought to be involved in synaptic plasticity in hippocampus [17,18,19], it also plays versatile roles on behaviors by affecting neural transduction such as anxiety and depression in diabetic rats [20,21]. It has been emphasized that the anxiety- and depression-like behaviors are correlated with serum leptin levels in non-stressed rat model. Acute administration of leptin not only induces weight loss [3,22], but also ameliorates anxious and depressive effects [23,24]. Leptin level in plasma is found to be correlated with emotional status [25].

### 1.3. Fish Is a New Low Vertebrate Model for Leptin-Associated Study

Numerous mammalian models have contributed to leptin studies [26,27,28]. Based on previous genomic project studies, leptin in tetrapods shows closer relationship with cartilaginous fish than with the bony class [28]. In humans, leptin binds to specific leptin receptor, LepR, which has several isoforms, and mediates different signal pathways associated with dietary and energetic homeostasis, such as Janus Kinase-Signal Transducer and Activator of Transcription-s (JAK-STAT3), Phosphatidylinositol 3-Kinase (PI3K), Mitogen-activated Protein Kinase (MAPK), and 5’Adenosine Monophosphate-activated Protein Kinase (AMPK) [29,30,31].

Fish is a new animal model for functional research for leptin among lower vertebrates. In fish, two leptins (A and B) are encoded from multiple *Ob* genes that interact with LepR family [32,33]. Both leptin and LepR-knockout model in zebrafish show promise in studying leptin-associated behaviors with leptin/LepR hormone system being evolutionarily conserved between fish and mammals [34]. In addition, leptin and LepR gene deficiencies display an increased food uptake and metabolism, which is similar to dysfunction exhibited in other fasting animal models [33]. In addition, adipogenesis has been well studied in zebrafish model, which greatly facilitates research on obesity [34]. Moreover, the genetic manipulation tools for loss-of-function analysis (TALEN and Clustered Regularly Interspaced Short Palindromic Repeats, CRISPR, genome editing system) [35,36,37] and gain-of-function analysis (i.e., Tol2 transposon system) [38,39] are well developed in fish. The behavioral assays in fish have been significantly improved and standardized during the past years [40,41]. Combining advantages mentioned above, fish is an ideal model for performing behavioral assay in a leptin loss-of-function study.

The aim of this study was to use zebrafish as a model to validate the potential function of leptin gene especially on behavior. Compared to previous studies in rodents, we performed analyses in leptin a (*lepa*) knockout (KO) zebrafish with novel tank, mirror biting, predator avoidance, social interaction, shoaling, color preference and circadian rhythm tests for multiple behavioral endpoints such as anxiety, new environment exploration, aggressiveness, fear response, social behavior, day/night activity in circadian rhythms and color preference assays. With the observation of the behavioral abnormalities in *lepa* KO fish, a better understanding of the role of leptin in physiological and behavioral regulations was confirmed.

## 2. Results

### 2.1. Lepa KO Zebrafish Displayed Typical Obesity Phenotype

**Figure 1:**
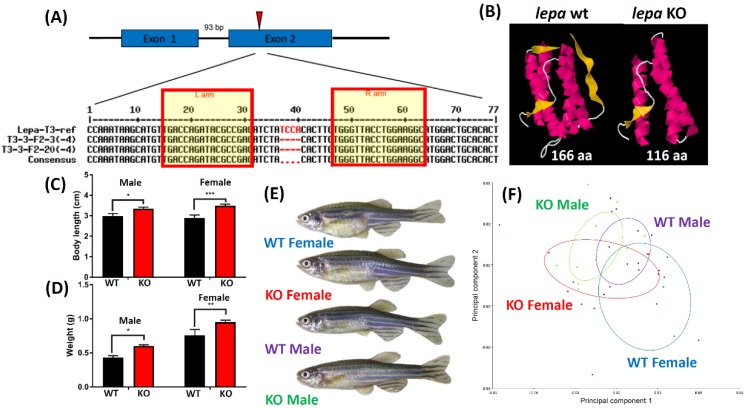
Generation and phenotype analysis of *lepa* KO zebrafish. (**A**) Schematic showing the TALEN left and right arm sequences used to target *lepa* gene on exon 2 in zebrafish. (**B**) Three-dimension model showing the predicted protein structure between wild type and *lepa* KO fish. (**C**) Comparison of body length and (**D**) body weight between wild type and *lepa* KO fish at six-month-old aged. (**E**) Appearance of wild type and *lepa* KO fish at six-month-old aged. (**F**) Morphometric analysis of wild type and *lepa* KO fish at six-month-old aged.

There are two leptin gene duplicates in zebrafish genome. The leptin a (*lepa*) gene in zebrafish genome is located on chromosome 18 and contains two exons, while the leptin b (*lepb*) gene is located on chromosome 4 and contains two exons. Based on phylogenetic tree topology, fish *lepa* is closet to *lepb* than their mammalian *lep* counterpart [42]. To generate *lepa* KO zebrafish, the 5’-capping in vitro synthesized TALEN on the left and the right arms of mRNAs targeting exon 2 were injected into embryos (Figure 1A). The injected embryos were raised to adulthood, and mated with wild-type fish to generate F1 progenies. The potential mutant carriers were screened and sequenced before crossing the F1 pairs with the same genotype to generate homozygotic F2 progenies. Finally, one *lepa* mutant carrying 4bp deletion (TCCA deletion) was identified (Figure 1A). The corresponding translated protein sizes for mutated *lepa* gene was shorten from 166 amino acid (aa) to 116 aa. Three-dimensional modeling shows the mutated *lepa* protein is truncated with only two beta-sheet and one alpha-helix motifs at the C-terminus (Figure 1B). When fish reached sex maturation at nine months old, *lepa* KO fish displayed obese phenotype within their body length (Figure 1C) and body weight (Figure 1D), significantly larger than the wild type (WT) fish in both genders. The *lepa* KO fish also had better appetites than control fish (data not shown). Morphometric measurements were taken by labeling several key landmarks according to those previously published [43]. Although *lepa* KO fish displayed obese phenotype, principle component analysis (PCA) showed that the *lepa* KO and WT fish exhibited no significant overall change on fish shape (Figure 1E,F).

### 2.2. Lepa KO Zebrafish Display Hyperactivity with Faster Locomotion and Robust Exploratory Behaviors

**Figure 2:**
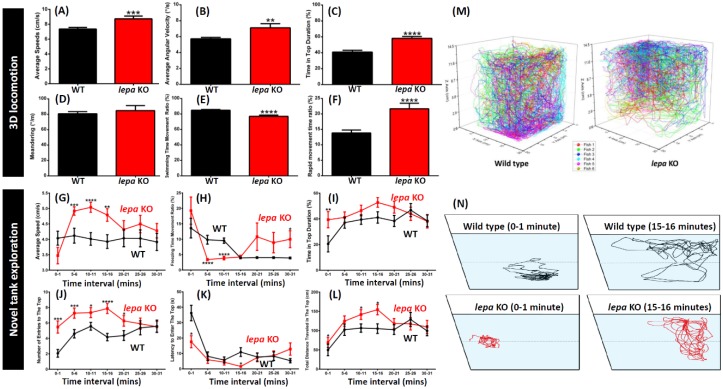
Comparison of several untreated wild type and *lepa* KO zebrafish behavioral endpoints in 3D locomotion test and novel tank exposure test within 30 min: (**A**) Average speed; (**B**) average angular velocity; (**C**) duration time in top of the tank; (**D**) meandering; (**E**) swimming time movement ratio; (**F**) rapid movement time ratio; (**G**) average speed; (**H**) freezing time movement ratio; (**I**) duration time in top of the tank; (**J**) number of entries to the top of the tank; (**K**) latency to enter the top of the tank; and (**L**) total distance travelled in the top of the tank. (**M**) Locomotion trajectory of wild type (left) and *lepa* KO (right) zebrafish. The three-dimensional locomotion trajectories of six fish were recorded and analyzed by using idTracker software. (**N**) Locomotion trajectory of wild type (black color) and *lepa* KO (red color) zebrafish in novel tank test. The locomotion for 0–1 min (left) and 15–16 min (right) windows were recorded and analyzed by using idTracker software. The data from 3D locomotion test (**A**–**F**) are expressed as the mean ± SEM and analyzed by unpaired t-test (*n* control = 40; *n lepa* KO = 30; ** *p* < 0.01, *** *p* < 0.001, **** *p* < 0.0001). The data from novel tank test (**G**–**L**) are expressed as the mean ± SEM and analyzed by Mann–Whitney test (*n* = 30; * *p* < 0.05, ** *p* < 0.01, *** *p* < 0.001, **** *p* < 0.0001).

In addition to canonical function in obesity, the potential function of *lepa* gene in behaviors by measuring multiple behavioral endpoints were investigated. Firstly, we tested the three-dimensional (3D) locomotion activity (a test for measuring fish locomotion in three-dimensional space) of the *lepa* KO fish according to our previously published protocol [44]. After 15 min acclimation in the experiment tank, the *lepa* KO mutant fish had higher locomotion activity than the control fish, which was manifested in a higher average speed (*p* < 0.001) (Figure 2A) and a higher average angular velocity (*p* < 0.01) (Figure 2B). In addition, higher swimming movement, in terms of swimming time movement ratio, and rapid movement time ratio, was also observed in the *lepa* KO fish (*p* < 0.0001) (Figure 2E,F). Furthermore, a more robust exploratory behavior was exhibited by *lepa* KO fish, which was quantified by longer time duration spent in the top portion of the tank compared to the control fish (*p* < 0.0001) (Figure 2C). The locomotion trajectory tracking can be found in Figure 2M (also see Video S1). There was no difference for movement without a fixed direction or path for both the wild type and *lepa* KO fish, as demonstrated by similar meandering values (Figure 2D).

During the novel tank exposure test (a test for measuring the anxiety level of fish facing a novel environment), after 5 min of acclimation, the *lepa* KO mutant fish showed a significantly higher locomotion activity, a higher average speed (*p* < 0.001), and a lower freezing time movement ratio (*p* < 0.0001) (Figure 2G,H). The high level of locomotion activity was maintained during the first 20 min of novel tank exposure and gradually subsided afterwards, compared to the wild type fish behavior (Figure 2G,H). The *lepa* KO fish also exhibited more robust traits of the aforementioned behaviors than the control fish during exploratory behavior observation, namely longer duration spent at the top of the tank, higher number of entries to the top portion of the tank, and longer travelling distance at the top of the tank, as well as lower latency to enter the top of the tank (Figure 2I–L). The locomotion trajectory tracking can be found in Figure 2N.

### 2.3. Lepa KO Zebrafish Displayed Less Aggression and Less Fear to Predator

**Figure 3:**
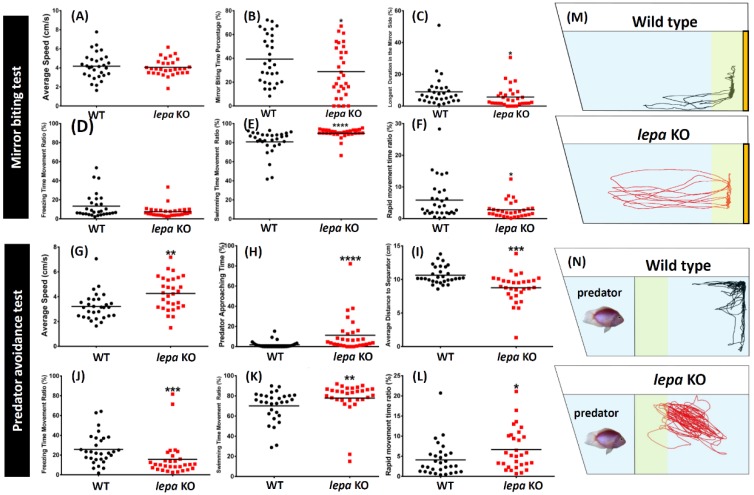
Comparison of several behavior endpoints of untreated wild type and *lepa* KO zebrafish in mirror biting test (**A**–**F**) and predator avoidance test (**G**–**L**): (**A**) average speed; (**B**) mirror biting time percentage; (**C**) longest duration in the mirror side; (**D**) freezing time movement ratio; (**E**) swimming time movement ratio; (**F**) rapid movement time ratio; (**G**) average speed; (**H**) predator approaching time; (**I**) average distance to separator; (**J**) freezing time movement ratio; (**K**) swimming time movement ratio; and (**L**) rapid movement ratio. (**M**) Locomotion trajectory of wild type (black color) and *lepa* KO (red color) zebrafish in mirror biting test. The mirror positions are positioned to the right site. The mirror approaching zones are highlighted in yellow color. (**N**) Locomotion trajectory of wild type (left) and *lepa* KO (right) zebrafish in predator avoidance test. The predator is placed in the left side and zebrafish is placed in the right site and one transparent glass plate is inserted to separate both animals. The predator approaching zones are highlighted in yellow color. The data are expressed as the mean ± SEM and were analyzed by Mann–Whitney test (*n* = 30; * *p* < 0.05, ** *p* < 0.01, *** *p* < 0.001, **** *p* < 0.0001).

To investigate aggressive behavior in the *lepa* KO zebrafish, we performed mirror biting test (a test for measuring the aggressive level of fish to interact with their own image in the mirror). We found that the *lepa* KO mutant zebrafish displayed significantly less aggression. The *lepa* KO mutant showed lower mirror biting time percentage and longer duration in the mirror side compared to the wild type control fish (*p* < 0.05) (Figure 3B,C). The locomotion trajectory tracking can be found in Figure 3M (Video S3). Furthermore, the *lepa* KO fish did not show any significant difference in their locomotion activity, as indicated by a similar average speed (Figure 3A). However, different distribution of zebrafish movement was noted in the mutant fish with higher swimming time movement ratio (*p* < 0.0001) (Figure 3E) and lower rapid movement ratio (*p* < 0.05) (Figure 3F). Meanwhile, there was no difference of freezing time movement ratio between these fish (Figure 3D).

In the predator avoidance test (a test for measuring the fear level when fish face their predator), less fear response within *lepa* KO mutant fish was observed when predator fish was placed into the experiment tank. This phenomenon was corroborated by several altered anti-predatory avoidance behavioral endpoints, including higher predator approaching time (*p* < 0.0001) (Figure 3H) and lower average distance to the separator between the predator fish (*p* < 0.001) (Figure 3I). Furthermore, higher locomotion activity of *lepa* mutant fish in this predator avoidance test was also observed, which showed a higher average speed (*p* < 0.01) (Figure 3G). Different distribution of zebrafish movement types, namely short freezing time movement ratio (*p* < 0.001) (Figure 3J), longer swimming time movement ratio (*p* < 0.01) (Figure 3K), and rapid movement ratio, were also noted (*p* < 0.05.) (Figure 3L). The locomotion trajectory tracking can be found in Figure 3N (also see Video S4).

### 2.4. Lepa KO Zebrafish Displayed Less Social Interaction and Loose Shoaling

**Figure 4:**
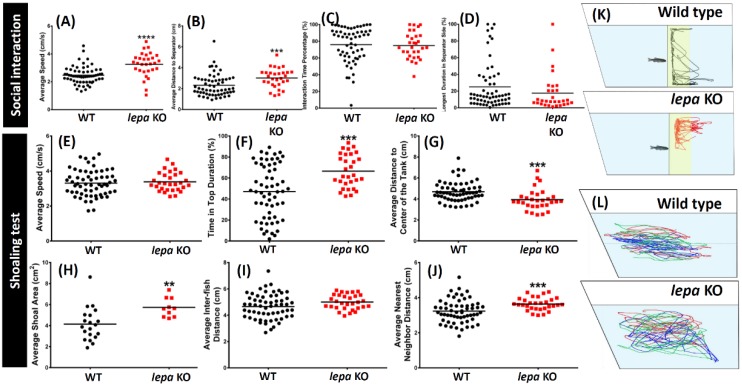
Comparison of several behavior endpoints of untreated wild type and lepa KO zebrafish in social interaction test (**A**–**D**) and shoaling test (group of three fish) (**E**–**J**). (**A**) average speed; (**B**) average distance to separator; (**C**) interaction time percentage; (**D**) longest duration in separator side; (**E**) average speed; (**F**) time in top duration; (**G**) average distance to center of the tank; (**H**) average shoal area; (**I**) average inter-fish distance; and (**J**) average nearest neighbor distance. (**K**) Locomotion trajectory of wild type (top) and *lepa* KO (bottom) zebrafish in social interaction test. The normal zebrafish is placed in the left side and tested KO fish is placed in the right site and one transparent glass plate is inserted to separate both animals. The social interaction approaching zones are highlighted in yellow color. (**L**) Locomotion trajectory of three wild type (top) and *lepa* KO (bottom) zebrafish in shoaling test. The shoaling trajectories for each single fish are labeled in different color. The data were expressed as the mean ± SEM and were analyzed by Mann–Whitney test (*n* = 30; ** *p* < 0.01, *** *p* < 0.001, **** *p* < 0.0001).

In the social interaction test (a test for measuring the social interaction to approach and remain near conspecifics), *lepa* KO zebrafish showed slightly disrupted ability of interacting with conspecifics. Our results show high locomotion activity, longer average distance to separator (*p* < 0.001), and long duration to separator side in *lepa* mutant fish. Together, these data suggest abnormal social interaction with conspecifics (Figure 4A,B,D). The locomotion trajectory tracking can be found in Figure 4K (also see Video S5). However, similar interaction time percentage were recorded (Figure 4C).

Furthermore, deficiency of *lepa* gene also caused the mutant fish to form different shoaling behavior in shoaling test (a test for measuring the social interaction of multiple zebrafish). We tested the shoaling behavior of three fish and found a loose shoal was formed by the *lepa* KO fish with larger average shoal area and average nearest neighbor distance (Figure 4H,J). The locomotion trajectory tracking can be found in Figure 4L (also see Video S6). Nonetheless, no significant difference between the control and mutant fish average inter-fish distance was observed (Figure 4I). Consistent with the results from another test (i.e., 3D locomotion and novel tank test), *lepa* KO fish showed more noticeable top-bottom exploratory behavior with longer time at the top of the tank (Figure 4F). Interestingly, less horizontal exploratory behavior in mutant fish was observed (Figure 4G). The locomotion activity for both fish in this shoaling test was at a similar average speed level and showed no difference (Figure 4E).

### 2.5. Lepa KO Zebrafish Display Dysregulation in Circadian Rhythm

**Figure 5:**
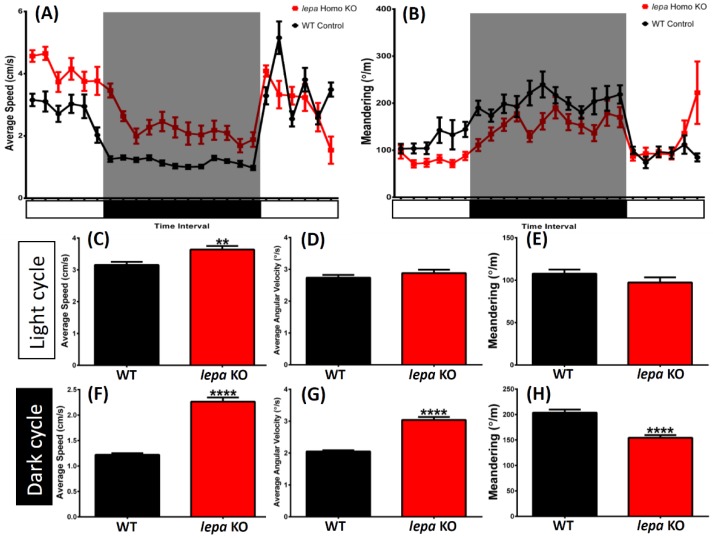
Comparison of several untreated wild type and *lepa* KO zebrafish behavior endpoints in circadian rhythm test: (**A**) circadian patterns of average speed; and (**B**) meandering in light/dark cycle. (**C**) Average speed; (**D**) average angular velocity; and (**E**) meandering during light cycle. (**F**) Average speed; (**G**) average angular velocity; and (**H**) meandering in during dark cycle. The data are expressed as the mean ± SEM and several data were analyzed by unpaired t-test (*n* control = 30; *n lepa* KO = 16; ** *p* < 0.01, **** *p* < 0.0001).

During circadian rhythm test with Light/Dark (LD) 12/12 h setting, *lepa* KO zebrafish generally maintained higher locomotion activity than the control fish most of the time with higher average speed and less meandering within 24 h (Figure 5A,B). Furthermore, when compared the locomotion activity in the light and dark cycles, respectively, higher locomotion activity was observed among the *lepa* mutant fish during the light cycle compared to the wild type fish. Higher average speed with higher average angular velocity and slightly less meandering was observed in the *lepa* KO fish. However, the differences were not statistically significant with the control (Figure 5C–E). As expected, during the dark cycle, the *lepa* KO fish showed a significantly higher average speed, a higher average angular velocity, and significantly less meandering compared to the wild type fish (Figure 5F–H)

### 2.6. Lepa KO Zebrafish Displayed Reduction of Color Preference

**Figure 6:**
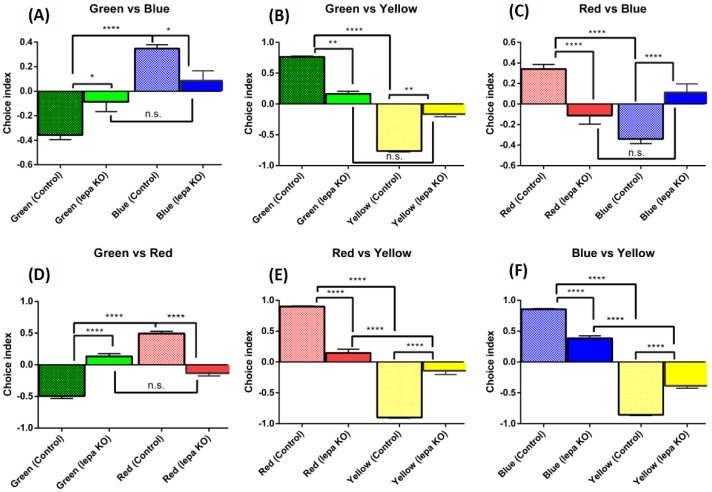
Comparison of color preferences between wild type and *lepa* KO zebrafish: (**A**) green vs. blue combination; (**B**) green vs. yellow combination; (**C**) red vs. blue combination; (**D**) green vs. red combination; (**E**) red vs. yellow combination; and (**F**) blue vs. yellow combination. Data were analyzed using two-way ANOVA followed by Tukey post-hoc test. If the data were not normally distributed, they were analyzed using non-parametric Kruskal–Wallis followed by Dunn’s post-hoc test, and *p* < 0.05 was considered significantly different. The data are presented with mean ± SEM with n = 24, * *p* < 0.05, ** *p* < 0.01, *** *p* < 0.001, **** *p*> 0.0001.

Since *lepa* KO fish display less aggression on mirror biting assay, we wondered whether the *lepa* KO fish would also show less general interest in other issues. We tested this hypothesis by performing color preference test. For normal condition, the wild type fish displayed normal color preference in the following sequence of: red > blue > green > yellow. However, the color preference test showed that the *lepa* KO zebrafish have different color preferences compared to the wild type zebrafish (Figure 6). Overall, their color preferences were reduced for all color combinations and the preference shifted to: blue = green = red > yellow. In green and blue combination, the wild type preferred blue color (*p* < 0.0001), but in *lepa* KO fish the result showed there was no preference between blue or green (*p* > 0.2048) (Figure 6A). In green and yellow combination, the wild type preferred the green color (*p* < 0.0001) and *lepa* KO fish showed no preference for green or yellow (*p* > 0.1638) (Figure 6B). Wild type zebrafish showed red color preference in red and blue combination (*p* < 0.0001), but the *lepa* KO fish showed no preference for either color (*p* > 0.0895) (Figure 6C). The green and red combination test showed red preference in the wild type (*p* < 0.0001), but no preference for *lepa* KO fish (*p* > 0.0558) (Figure 6D). The red and yellow combination test showed the wild type and *lepa* KO fish still preferred the red color over yellow color, but their preference intensity was reduced significantly (*p* < 0.0001) (Figure 6E). The blue and yellow combination test also showed wild type and *lepa* KO fish prefer blue color over yellow color and the intensity also reduced significantly (*p* < 0.0001) (Figure 6F).

### 2.7. Lepa KO Zebrafish Display Normal Short-Term Memory

**Figure A1:**
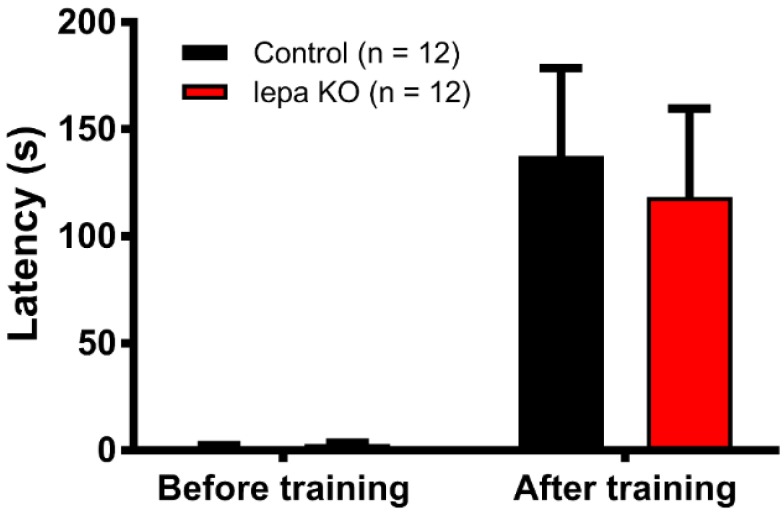
Passive avoidance test on training and memory of control (black) and *lepa* KO (red) zebrafish. Data were presented as means ± standard error of the mean (SEM) (*n* = 12) and were analyzed individually for each treated group. Statistical analysis was performed by one-way ANOVA, followed by Tukey post hoc test.

In rodents, the obese rat triggered by high fat diet shows spatial memory impairment [45]. The impairment of long-term potentiation and spatial memory in leptin receptor-deficient rat has been reported [46]. In addition, the administration of leptin can reduce pathology and improve memory in a transgenic mouse model of Alzheimer’s disease [47]. This combinational information suggests leptin might also play a role in memory in zebrafish. We tested this hypothesis by performing passive avoidance assay to test the short-term memory on avoiding the electric shock in the dark chamber. Results show both control and *lepa* KO fish display similar latency or retention time on moving to the punishment compartment (black box) on both training and testing session, indicating the short-term memory was maintained at same level between control and *lepa* KO fish (Figure A1). This result indicates that *lepa* KO fish display similar short-term memory level with wild type fish.

### 2.8. Dysregulation of Appetite-Controlling Hormones in the Lepa KO Fish

The multiple behavioral abnormalities in *lepa* KO fish prompted us to address the underlining mechanism. By performing ELISA with antigen-specific antibodies, we quantitatively compared the neurotransmitters and other biochemical markers between control and *lepa* KO fish (Table 1). We used creatinine as an internal control marker due to its stable production rate. For obesity-related phenotype, first, we measured the leptin content in brain and muscle by ELISA. Results show that the leptin content was about two- and four-fold reduced in brain and muscle tissues, respectively, in the *lepa* KO fish. Leptin is secreted from peripheral adipose tissue and then feeds back target to the leptin receptor in the brain. For leptin receptor, we found it was two-fold upregulated in *lepa* KO fish brain, suggesting this upregulation of leptin receptor might function to compensate for the loss of leptin in *lepa* KO fish. For AgRP and Ghrelin, as two important hormones in stimulating appetite, we found their levels were about two- and four-fold upregulated in *lepa* KO fish brain. In addition, we also found two-fold upregulation fofor glucose and two-fold reduction of insulin levels in *lepa* KO fish brain. This result suggests the *lepa* KO fish failed to maintain constant levels of glucose and insulin compared to the control fish.

### 2.9. Dysregulation of Neurotransmitter in the Lepa KO Fish Brain

For behavior alteration issue, we measured seven major brain neurotransmitters, including serotonin, norepinephrine, glycine, dopamine, histamine, acetylcholine and melatonin, by ELISA. Our results show a 12-fold reduction in both serotonin and norepinephrine levels in *lepa* KO fish brain. Serotonin is known to suppress appetite [48,49,50]. Our data in *lepa* KO fish were consistent with the findings that induction of depression and greatly enhancement on appetites in higher vertebrates when serotonin level is reduced [51,52,53]. Norepinephrine is a hormone controlling mobilization between brain and muscle. Consistent with previous study demonstrating that animals show depression and lack of motivation due to low level of norepinephrine [54,55], we found that the low level of norepinephrine was expressed in *lepa* KO fish, which showed reduced aggression and predator avoidance behaviors. In addition, elevation of glycine and dopamine in *lepa* KO fish brain was observed. The elevation of dopamine in *lepa* KO fish confirmed that leptin signal plays a role in controlling dopamine-associated system to suppress feeding [56,57]. Dopamine elevation in *lepa* KO fish may trigger the signal of food cravings (by showing hyperactivity) beyond necessary and therefore weight gain ensued. The hyperactivity in *lepa* KO fish led us to ask whether this intriguing phenotype was contributed from high energy storage in the muscle. By using ELISA, we found the creatine kinase and ATP levels were four- and three-fold reduced in *lepa* KO fish muscle. This result indicates the energy storage in the muscle was greatly reduced in *lepa* KO fish.

Although we did not identify memory impairment in the *lepa* KO fish, a great reduction for the ACh (acetylcholine), a major memory-related neurotransmitter, was detected in *lepa* KO fish brain. Other markers for Alzheimer’s disease such as amyloid Aβ and p-Tau showed no difference between wild type and *lepa* KO fish. In *lepa* KO fish, we found the circadian rhythm was dysregulated by showing very high locomotion activity during both day and night cycles. By ELISA, we confirmed the sleeping control hormone of melatonin was 12-fold downregulated in *lepa* KO fish brain, suggesting dysregulation of circadian rhythm in *lepa* KO fish was due to low level of melatonin.

### 2.10. Leptin Deficient Zebrafish Exhibited Greater Oxidative Damage in the Brain

The enunciated anxiety and hyperactivity in *lepa* KO fish led us to hypothesize that the stress hormone levels in the brain tissues were elevated. To this end, we measured the catecholamine and cortisol levels by ELISA. The results show that the level of catecholamine was three-fold decreased in *lepa* KO fish, while the level of cortisol in *lepa* KO fish brain was 1.5-fold increased. The intracellular ROS (reactive oxygen species) level was two-fold elevated and the anti-oxidative stress enzyme of catalase level was 22-fold reduced in *lepa* KO fish. In line with the body of literature suggesting a direct regulatory role of reactive ROS on HIF-1α [58,59], the level of HIF-1α was found to be significantly elevated in muscle tissues of the leptin deficient fish. Oxidative stress and inflammation are concatenated processes that are usually activated in cells due to stress [60]. However, we found that TNF-α, IL1β and histamine showed no significant difference in *lepa* KO fish brain compared to the wild type fish brain.

## 3. Discussion

Leptin is a molecular link between fat and the brain. In addition to its canonical role on appetites control, in this study, we provide direct in vivo evidence to support that *lepa* gene plays important roles in behavior control such as anxiety, aggressiveness, fear and circadian rhythm in zebrafish. By ELISA, we also provide biochemical data showing the dysregulation of neurotransmitters (i.e., serotonin, norepinephrine and dopamine) in the brain of *lepa* KO fish. Recent works have demonstrated direct leptin effects on dopamine neuron function and behavior, in which peripheral hormones influence behaviors and contributes to a more comprehensive model of neural control over food intake [61,62,63,64]. The lack of control over food intake bears resemblance to drug addiction, where loss of control over behavior leads to compulsive drug use. In addition to leptin, serotonin and dopamine regulate the feeding behavior and metabolic processes that involve energy expenditure through their effects on the activity of the pro-opiomelanocortin (POMC)-expressing neurons of the hypothalamic arcuate nucleus (ARC) [65,66,67,68].

### 3.1. Leptin and Leptin Receptor Mutant Fish

Two leptin genes and one leptin receptor gene have been identified and characterized in zebrafish genome. In previous studies, by using genome editing approach, zebrafish mutant lines carrying *lepa, lepb,* or *lepR* gene deficiency have been generated [69]. However, unexpectedly, there was only slightly pancreaticβ-cell hyperplasia with no obese phenotype reported in all three zebrafish mutant lines. However, in medaka fish, leptin receptor deficiency leads to obesity [70]. In this study, we reported a zebrafish mutant line carrying *lepa* gene deficiency that displayed a typical obesity phenotype showing faster growth rate, obese, higher appetites and appetites-controlling neuropeptide dysregulation. The discrepancy in *lepa* KO phenotype reported by different research groups might be due to the different alleles that have been mutated. Similar phenomenon has been reported in human patients showing different degrees of obesity by carrying different mutated leptin genotypes [71]. The *lepa* KO fish reported in this study provides an excellent and better model for obesity study in the future.

### 3.2. Behavior Alterations in Lepa KO Fish

In addition to obesity, we reported *lepa* KO zebrafish displayed hyperactivity, altered aggressiveness, social interaction, and predator avoidance behaviors for the first time. These findings provide strong evidence for a critical role of *lepa* gene on modulating anxiety-like behaviors in zebrafish, which has been demonstrated by pharmacological studies in rat models showing leptin has anti-depressant-like properties [72]. Diabetic rats expressing low level of leptin show hyperactivity behavior with increase of total movement time, and high anxiety levels than the control group in elevated plus maze test [73]. In zebrafish, hyperactivity or hyperlocomotion has been well addressed as an anxiety-related behavior and related to psychostimulant/convulsant action [73].

The aggression behavior is generally controlled by multiple hormones in the brain, such as neuropeptide hormone oxytocin and several classes of steroid hormone, including androgens and estrogen [74]. In addition, the primary hormone involved in aggressive behavior is the sex hormone testosterone [75]. However, whether leptin plays a role in aggression is rarely investigated. Recent studies reveal that high fat diet and high cholesterol diet cause Göttingen minipigs to become less aggressive and less fearful [76]. However, the leptin level has not been analyzed in this diet-induced-obesity (DIO) minipig model. Our model provides direct evidence that link *leptin* gene function to aggression using a well-established paradigm of mirror biting that is traditionally used for fish aggressive behavior studies [77]. Our result also showed that the *lepa* KO mutant fish exhibit similar behavioral deficits in social interaction test, a test to assess zebrafish sociability by observing the interaction between fish and its conspecifics [77]. The anxiogenic effect of loss of *lepa* gene was corroborated by our finding that slightly increase in average distance to separator between the mutant fish and conspecifics (Figure 3). This phenomenon was observed during the test and might be related to the hyperactivity behavior that they showed during this test. In mouse, leptin’s effects on social interaction have been reported. In agreement with our result, acute administration of leptin at 1 mg/kg reveals that distinct social behavioral components are modulated, as indicated by the increased total time of active social behaviors [77].

A related social behavior, shoaling behavior, is common in many species of fish representing the complex interactions of animals moving together in coordinated movements. This behavior is related to foraging, mating, fear response, and defense against predators [78]. Previously, it has been shown, in zebrafish, that alcohol and nicotine cause anxiolytic behavior, which results in a significantly loosened shoal [79]. In addition, hallucinogenic drug lysergic acid diethylamide (LSD) evoke hallucinogenic and/or anxiolytic-like effects, with disrupted shoaling and increased average inter-fish distance [76]. To shoal, fish must prefer to approach and remain near conspecifics. Behavioral deficits in social interaction of the *lepa* KO mutant fish may result in loose shoals forming with increased average shoal area and average nearest neighbor distance. Taken together, deficiency of *lepa* gene in zebrafish showed loose shoal, indicative of social withdrawal behavior in *lepa* mutant fish.

Fear, as a response to imminent threat, has been studied as reactions to alarm pheromone and predators in zebrafish [80]. Leptin has been identified to play an anxiolytic function by facilitating fear extinction in rat model using a gain-of-function assay [81]. In this study, we provide direct evidence of the role of *lepa* gene in fear extinction. In our predator avoidance test, *lepa* KO mutant fish generally did not respond to predatory species with fear reactions and preferred to spend more time close to a predator fish. This interpretation was corroborated by the low level of catecholamine, a stress hormone, found in the mutant fish brain. On the other hand, fat accumulation in body often is associated with an increased risk of predation. Moving phylogenetically closer to humans, it is obvious that excess body fat is not favorable in primates whose defenses against predation depends on the ability to move and the speed of movement [81,82]. In addition, fear response of zebrafish also includes forming stronger shoal cohesion [80]. Therefore, loss of fear response in *lepa* KO mutant fish could also be a reason for the loose shoal observed in the shoaling test in the present study.

Leptin fluctuation in serum is influenced by circadian rhythm. The leptin level is relatively low during daytime and is high at night [83]. Circadian rhythm disruption leads to leptin dysfunction, implicating the central clock in body weight control [84]. However, little is known about how *leptin* gene plays a role in zebrafish circadian rhythm control. Here, we showed a novel finding indicating circadian rhythm deregulation in *lepa* KO fish. It has been shown that obese rats exhibited abnormalities in circadian-related patterns, such as equal distribution of slow-wave sleep and paradoxical sleep throughout the light–dark cycle, abnormality of thyroid-stimulating hormone level, and no circadian rhythms of activity after 30 weeks of age. The abnormal circadian variations in sleep, thyroid-stimulating hormone level and corticosterone seen in mutant rats suggest the possibility of a neural deficit in the circadian timing system [85]. Kohsaka and colleagues also showed that a high-fat diet in mice led to changes in the period of the locomotor activity rhythm and alterations in the expression and cycling of canonical circadian clock genes [86]. Taken together, these results indicate that consumption of a high-calorie diet alters circadian clock in mammals. Melatonin has been reported to have direct effect on inducing sleep in human [87]. The biochemical assay showed the lower expression of melatonin may cause dysregulation of circadian rhythm in zebrafish. Therefore, high activity of *lepa* KO zebrafish at night may be attributed to low melatonin level.

Color vision is one of the most important modalities in the recognition of biologically important stimulation, and thus it plays a critical role in visual perception. The *lepa* KO fish showed distinct color preference pattern compared with the wild type fish, with the *lepa* KO fish losing the preferences for red, green and blue color. Similar to wild type fish, the *lepa* KO fish also avoided yellow color, suggesting that the *lepa* KO fish was still able to distinguish different colors. The color preference of depression induced zebrafish was similar to that of the *lepa* KO fish pattern found in the present study, with no preference among blue, red and green color [88]. It has been reported that humans with depression are likely to choose neutral colors such as grey and black compared with healthy humans who would choose blue and red color [89]. Together, these results lend further support for the notion that *lepa* mutation may lead to cognitive disorder and depression. Due to its relative simplicity and ease, color preference screening using zebrafish is suitable for high-throughput screening applications. This system may potentially be applied to the analysis of drug effects on zebrafish behavior or the detection of sensory deficits in neurological disorder models, such as autism-related disorders.

Serotonin is believed to influence a variety of psychological conditions and has been implicated in cognition, mood, anxiety and psychosis. This includes brain cells related to mood, sexual desire and function, appetite, sleep, memory and learning, temperature regulation, and some social behaviors [90]. Imbalance in serotonin levels may influence mental condition that leads to depression [91,92]. In this study, the most significant finding is the great reduction of serotonin level in *lepa* KO fish brain. The low level of serotonin in *lepa* KO fish brain might be due to low brain cell production of serotonin, a lack of receptor sites able to receive the serotonin that is made, inability of serotonin to reach the receptor sites, or a shortage in tryptophan, and need further studies to clarify this hypothesis. Certain genetic variations may predispose people to develop anxiety disorders. Another neurotransmitter associated with anxiety is norepinephrine, which also showed great reduction in *lepa* KO fish model. The serotonin system has reciprocal interactions with the norepinephrine system. Patients with anxiety disorders such as panic disorder have increased norepinephrine reactivity and/or tone. In addition, the low norepinephrine level in *lepa* KO fish may reduce the aggressive response by showing less fear response in predator avoidance test.

### 3.3. Possible Mechanism for Behavior Alterations in Lepa KO Fish

**Figure 7:**
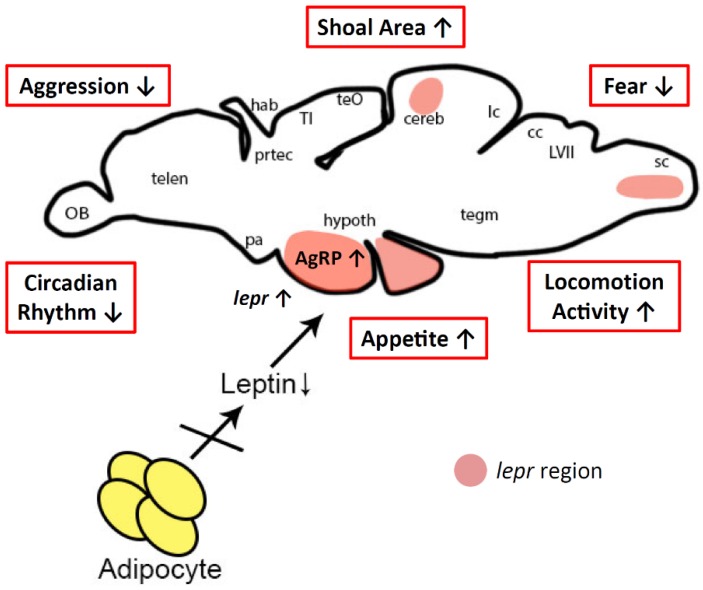
Proposed mechanism of leptin deficiency that affected on zebrafish behaviors. Leptin production by adipocyte is greatly reduced in *lepa*-deficient fish. Reduction of leptin induces the *lepr* overshooting in some specific regions on the zebrafish brain. Lack of leptin affects behavioral changes by deregulating HPA-axis and/or medulla oblongata functions resulting in dysregulated circadian rhythm, shoal formation, fear, and aggression, also increment of locomotion activity. Leptin deficiency also increases AgRP level, producing high level of appetite. OB, olfactory bulb; Telen, Telecephalon; TeO, Tectum Opticum; Hab, Habenula; Hypoth, Hypothalamus; SC, Spinal cord; Prtec, Pretectal population; Cc, Crista Cerebellaris; LVII, facial lobe; Tegm, tegmentum; Pa, Preoptic area; TI, Torus longitudinalis; Lc, Lobus caudalis.

Although the mechanisms for behavior alteration in *lepa* deficiency zebrafish remain speculative, high level of leptin receptor expressed in the brain and its substantial roles may be involved. The behavior changes are also suspected to be related with the depression behavior due to the *lepa* gene mutation that alter the serotonin and norepinephrine levels. Depression is reported to have a shared biological mechanism with obesity [93], which js influenced by food uptake and storage of energy that are known to be regulated by leptin. Interestingly, leptin has been reported to affect the development of the brain. Conceivably, leptin may affect the brain neurocircuitry and hypothalamic organization in early life [94]. Zebrafish leptin receptor (*lepr*) is detected in both of embryonic and larval stages, and in adult zebrafish. In embryonic zebrafish, *lepr* is mainly expressed in the notochord and it will decrease as development proceeds, while its expression in several other tissues including the trunk muscles and gut become evident [95]. Despite strong evidence showing *lepr* in other sites, the brain is the major sites that mediates the function of leptin. Inside the brain, the most abundant expression of *lepr* is exhibited by the hypothalamic lateral tuberal nucleus, the fish homolog of the arcuate nucleus. Meanwhile, outside the hypothalamus, *lepr* is also expressed in cerebellum, vagal lobe, hindbrain and other sites [95,96]. In general, leptin has pleiotropic effects, playing a key role in physiology including controlling various aspects of energy intake and expenditure, including centrally inhibiting appetite and suppressing lipogenesis, and influencing the stress response via the hypothalamo-pituitary-adrenal (HPA)-axis [97]. Major depression in human is often linked to deregulation of the endocrine stress system, particularly the HPA-axis [98,99]. HPA-axis is activated when the organism is exposed to a threatening stressor, and its stress adaptation response acts to maintain or restore the dynamic equilibrium (homeostasis) of body and mind [95]. In addition, the molecular and functional characteristics of the mammalian HPA-axis are highly conserved in zebrafish [100]. In zebrafish, HPA-axis is fundamental to stress responses, and involves a cascade of hormones from corticotropin releasing hormone (CRH) to adrenocortocotropic hormone (ACTH) and cortisol [101]. Our data support a significant higher cortisol level in *lepa* KO fish, which was consistent with previous study using mouse model showing elevated cortisol level in lack of leptin mice [102]. This finding is in line with previous study by Hewagalamulage et al. who highlighted that deregulation activity of the HPA-axis may not merely be a consequence of the obese state and high cortisol responsiveness affects an individual’s weight gain [103]. In parallel with our finding, the HPA-axis has complex interactions with serotonin, norepinephrine, and dopamine, which predicates that dysregulation of these hormones may cause mood disorder and depression [104]. Alternatively, expression of *lepr* (leptin receptor message) is also largely detected in the similar regions of the hindbrain (medulla oblongata) of both larval and adult zebrafish brains, which is similar to rodents. Furthermore, in five-day-old zebrafish larvae, the hindbrain *lepr*-expressing cell identity are already established, suggesting that the leptin-sensitive neural pathways are evolutionarily conserved [95]. The organization of hindbrain has elements of the same structural and functional network plan to the patterning in spinal cord and these two regions play a key role in generating all of the motor output from the nervous system [105]. In addition, previous study has shown hindbrain, together with the Mauthner cell and its serial homologs, serves to control the directionality of the escape behavior that fish use to avoid predators [106]. Although we established the correlation between *lepa* gene loss-of-function and behavioral abnormality, it is still unclear the underlying mechanism or neural circuit that is responsible for the observed behavior alteration in our *lepa* deficiency zebrafish model. It is certain that decreased leptin levels in *lepa* KO fish would modulate some region-specific brain function (as summarized in Figure 7). Future studies are considered necessary to address the expressional territories of leptin receptor or other neurotransmitters in the brain of *lepa* KO fish by using in situ hybridization or immunohistochemistry. In addition, some useful enhancer-trap transgenic lines showing neuron circuit-specific expression of green fluorescent protein (GFP) can be used to study the potential abnormality of neural circuit in *lepa* KO mutant [107,108,109].

In summary, by using loss-of-function assay approach, we report a *lepa* KO fish line showing obesity, hyperactivity, anxiety, less aggression and fear, and circadian rhythm and color preference dysregulation behaviors for the first time in zebrafish. This mutant model greatly facilitates gaining better insight into the non-canonical functions of leptin on behavior controls.

## 4. Material and Methods

### 4.1. Animal Ethics and Rearing

All experimental protocols and procedures involving zebrafish were approved by the Committee for Animal Experimentation of the Chung Yuan Christian University (Number: CYCU104024, issue date 21 December 2015). All experiments were performed in accordance with the guidelines for laboratory animals. Wild-type AB strain and *lepa* KO zebrafish were maintained in a recirculating aquatic system at 28.5 °C with a 10/14-h dark/light cycle according to standards. Circulating water in the aquarium was filtered by reverse osmosis (pH 7.0–7.5). The zebrafish were fed twice a day with lab-grown brine shrimp. For behavioral tests, we used adult zebrafish aged around 6–7 months.

### 4.2. Production of TALEN mRNA

The customized TALEN vectors were purchased from Zgenebio Inc. (Taipei, Taiwan). The TALEN target site was designed to target exon 2 of zebrafish leptin, which is located on chromosome 18 (ENSDARG00000091085). The TALEN target sequences TGACCAGATACGCCGAGatctatccacacttcTGGGTTACCTGGAAGGC (recognition sites are capitalized and underlined, and spacer is between two recognition sites). TALEN vectors were linearized with Not1 restriction enzyme and transcribed using mMESSAGE mMACHINE SP6 kit (Life Technologies) to synthesize 5’capping TALEN mRNAs. Following completion of transcription, poly(A) tailing reaction and DNase I treatment were performed according to the manufacturer’s instructions for the TALEN mRNAs. To enhance the purity, the in vitro transcribed TALEN mRNAs were then filtered and enriched by using YM30 column (Merck, Darmstadt, Germany).

### 4.3. Microinjection of Zebrafish Embryos

TALEN mRNAs were co-injected with Transposase mRNA into one-cell stage zebrafish embryos. Each embryo was injected with approximately 2 nL of solution containing 50 pg/nL of TALEN mRNA. On the next day, the injected embryos were inspected under a stereoscope. Only embryos that developed normally were used for analyses. Genomic DNA was extracted from embryos one day after injection for HRM (High Resolution Melting) assay.

### 4.4. Identification of Indel and Targeted Mutations by HRM Assay

Genomic DNA was extracted from pools of 10 embryos. Targeted genomic loci were amplified by using primers designed to anneal approximately 150–200 base pairs upstream and downstream from the expected FokI cut site and KOD FX high-fidelity DNA polymerase (TOYOBO) according to the manufacturer’s instructions. HRM assay was performed by using MyGO pro (IT-IS Life Science Ltd. Cork, Republic of Ireland). Each target locus was amplified by PCR from pooled genomic DNA of ten injected embryos. The resulting PCR products were cloned into the pGEM-T vector (Promega, Madison, USA) and transformed into bacteria. Thus, each colony represented one PCR amplified sequence.

### 4.5. Genotyping of Lepa KO Fish

At 3–4 days post-fertilization, 20 progenies were pooled and lysed in 25 mL of the alkaline lysis buffer (25 mM NaOH, 0.2 mM EDTA) and heated at 95 °C for 30 min. DNA solution was neutralized using 25 mL of the neutralization buffer (40 mM Tris-HCl, pH8.0). Samples were centrifuged at 3000 *g* for 5 min, and the supernatant contained extracted genomic DNA. In general, 20 embryos from each potential founder were screened for the presence of indel mutations by HRM amplifying the region surrounding the relevant TALEN FoKI cleavage site using a forward primer and a reverse primer. For sequence confirmation, genomic DNA from single embryos was amplified using targeted loci-specific primers. The PCR products were then submitted for Sanger sequencing. Mutant fish lines generated will be provided upon request.

### 4.6. Body Length and Body Weight Measurement

Mixed gender of wild type and *lepa* KO fish were raised in 10 L tanks separately until three months old (*n* = 60 for each group). Fish were transferred and raised in 100 L tanks. All the fish were feed with artemia twice per day and kept at constant temperature of 28 °C. The standard body length, body weight and survival rate were measured every month from 3 to 7 months old.

### 4.7. Zebrafish 3D Locomotor Activity

Three-dimensional locomotor activity was tracked according to a previously described protocol [45]. The 3D locomotion activity of six fish each recording time were held in an acrylic tank (20 cm × 20 cm × 20 cm dimension with 15 cm high water level).

### 4.8. Passive Avoidance Test (Short-Term Memory Test)

To evaluate the potential short-term memory impairment in *lepa* KO fish, the passive avoidance test was carried out according to previously published paper with minor modification [110]. The test included two sessions with an interval of 24 h. In each session, fish was placed individually in an acrylic tank (20 cm × 20 cm × 20 cm) divided by a door into black and white compartments with equal size. In the training session, the fish was placed in white compartment, with the door closed for one minute for environmental acclimatization and recognition. Next, the door was lifted, and the fish crossed over to the dark side of the tank. When fish passed the dark area, the door was again closed, and was subjected to a mild electric shock (25 V, 1 mA) of a 5 s interval. On the other hand, when fish passed the white area, fish was pushed back to the dark area and shocked again until the fish passed to the white region. The training was repeated for maximum of three attempts until the fish passed to the white side. The fish that completed the training was stored into small plastic container (15 × 10 cm × 5 cm) and kept at 28 °C. After 24 h, the fish was put into the training apparatus and tested for the latency to pass to the dark area. The latency observed in the test session was used as an indicator of short-term memory retention.

### 4.9. Aggressiveness Test

Aggressive behavior was determined in adult zebrafish based on the mirror test described before [111,112]. The test tank (28 cm × 5 cm × 15 cm) was filled with 1 L water where a mirror was at the side of the tank. After 1 min of fish introduction into the tank, aggressive behaviors (bites and fast swimming) were recorded for a period of 5 min.

### 4.10. Circadian Rhythm Test

To analyze zebrafish locomotion activity during day and night, we performed circadian rhythm test based on previous published method with some modifications [113]. To prevent any external disturbance, the tested fish were moved into a temperature-controlled incubator and kept at 28 °C. A specially designed LED light box with conventional and infrared LED array were used as bottom light source. An infrared sensitive CCD (700–1000 nm detection window) with maximum resolution at 1920 × 1080 pixels and 30 fps frame rate was used for video recording (3206_1080P module, Shenzhen, China). In this experiment, we recorded zebrafish locomotion activity (average speed, average angular velocity and meandering) for 1 min every hour and used idTracker software [114] to track fish movement trajectories according to our previous published method [45].

### 4.11. Predator Avoidance Test

The predator avoidance test was performed based on the mirror test described previously with minor modification [86]. The test tank (28 cm × 5 cm × 15 cm) was filled with 1 L water where a transparent separator was placed in the middle of the tank. The predator Convict cichlid (*Amatitlania nigrofasciata*) was placed in the left and zebrafish was put in the right area. After fish introduction into the tank, the predator avoidance behaviors (average speed, maximum and minimum speed, distance traveled, freezing, swimming, and rapid time movement percentage, top/bottom ratio of time spent and traveling distance, predator approaching time percentage, and distance to predator separator in average) were recorded for a period of 5 min.

### 4.12. Color Preferences Assay

The color preference assay was done in in a 21 cm × 21 cm × 10 cm acrylic tank filled with 1.5 L of filtered water. The tanks were divided into two-color combinations among red, green, blue and yellow, respectively. In total, there were six color combinations to determine the color preference of zebrafish. The experiment was done between 10:00 and 16:00. The color preference was recorded with combination of IR camera and HD camera and analyzed using idTracker software [114].

### 4.13. Determination of Appetite-Controlling Hormone, Neurotransmitter, Oxidative, Anti-Oxidative Capacity, Lipid Peroxidation, DNA Damage, Stress Hormone and Inflammation Markers

Three fishes were randomly collected from each tank (9 fish/treatment) for biochemical assay. Brains were removed and a pool of three zebrafish tissues were homogenated at medium speed with Bullet blender tissue homogenizer (Next Advance, Inc., NY, USA) with 50 volumes of (*v*/*w*) ice cold PBS adjusted to pH 7.2. Samples were further centrifuged at 12,000 *g* for 15 min and the crude homogenate was stored in 100 µL aliquots at −80 °C until further use. The levels appetite-controlling hormones of leptin, leptin receptor, AgRP, Ghrelin, glucose and insulin were measured by using target-specific ELISA kits purchased from commercial company (ZGB-E1642, ZGB-E1651, ZGB-E1647, ZGB-E1648, ZGB-E1649 and ZGB-E1650, Zgenebio Inc., Taipei, Taiwan). The levels neurotransmitters of serotonin, norepinephrine, dopamine, GABA, glutamate, glycine, ACh, AChE, catecholamine and cortisol were measured by using target-specific ELISA kits purchased from commercial company (ZGB-E1572, ZGB-E1571, ZGB-E1573, ZGB-E1574, ZGB-E1588, ZGB-E1587, ZGB-E1585, ZGB-E1637, ZGB-E1590 and ZGB-E1575, Zgenebio Inc., Taipei, Taiwan). The ROS and catalase levels were measured by using target-specific ELISA kits purchased from commercial company (ZGB-E1561 and ZGB-E1598, Zgenebio Inc., Taipei, Taiwan). Two stress hormones of catecholamine and cortisol were measured by using target-specific ELISA kits purchased from commercial company (ZGB-E1590 and ZGB-E1560, Zgenebio Inc., Taipei, Taiwan). Two inflammation markers of TNF-α and IL-1β were measured using target-specific ELISA kits purchased from commercial company (ZGB-E1612 and ZGB-E1608, Zgenebio Inc., Taipei, Taiwan). Initially, zebrafish brain tissues were minced and completely homogenized in PBS solution by using tissue homogenizer. The target protein content of each sample was calibrated by interpolation from the standard calibration curve and normalized to the amount of total protein (μg) in each sample. The target protein content or activity was measured following the manufactory instructions.

### 4.14. Statistical Analysis

Data for each fish group are expressed as mean ± SEM and were compared by using Student’s-*t* test. Significant level was set at 5% (*p* < 0.05). One-way ANOVA was used to analyzed color preferences data with Tukey post-hoc analysis. Non-parametric Kruskal–Wallis followed by Dunn’s post hoc test was used to measure data that did not follow normal distribution assumption (* *p* < 0.05, ** *p* < 0.01, *** *p* < 0.001, **** *p* < 0.0001). All statistics were plotted and compiled by using GraphPad prism (GraphPad Software version 7 Inc., La Jolla, CA, USA).

## Figures and Tables

**Table 1:** Comparison of expression levels of biochemical markers between untreated wild type and *lepa* KO zebrafish.

Biomarker	WT	*lepa* KO	Unit	Significance	*p* Value
**Brain**					
leptin	0.020 ± 0.002	0.010 ± 0.002	ng/μg total protein	YES	*p* = 0.0024
Leptin receptor	0.094 ± 0.012	0.213 ± 0.039	ng/μg total protein	YES	*p* = 0.0421
AgRP	0.145 ± 0.009	0.286 ± 0.021	ng/μg total protein	YES	*p* = 0.0036
Gehirn	0.118 ± 0.014	0.453 ± 0.058	ng/μg total protein	YES	*p* = 0.0051
Glucose	0.172 ± 0.020	0.358 ± 0.018	mmol/μg total protein	YES	*p* = 0.0024
Insulin	0.857±0.108	0.428 ± 0.098	U/μg total protein	YES	*p* = 0.0423
Serotonin (5-HT)	0.187 ± 0.034	0.0153 ± 0.003	ng/μg total protein	YES	*p* = 0.0076
Norepinephrine	0.731 ± 0.094	0.063 ± 0.010	ng/μg total protein	YES	*p* = 0.0021
Dopamine	5.530 ± 0.308	10.130 ± 1.138	pg/μg total protein	YES	*p* = 0.0175
GABA	0.020 ± 0.004	0.021 ± 0.005	ug/μg total protein	No	*p* = 0.8789
Glutamate	0.141 ± 0.007	0.053 ± 0.013	ug/μg total protein	YES	*p* = 0.0038
Glycine	0.239 ± 0.010	0.999 ± 0.170	ug/μg total protein	YES	*p* = 0.0110
ACh	6.150 ± 0.746	0.979 ± 0.118	U/μg total protein	YES	*p* = 0.0024
AChE	1.289 ± 0.159	0.541 ± 0.061	U/μg total protein	YES	*p* = 0.0117
Catecholamine	1.633 ± 0.086	0.589 ± 0.142	ng/μg total protein	YES	*p* = 0.0016
Cortisol	3.500 ± 0.325	5.155 ± 0.398	pg/μg total protein	YES	*p* = 0.0092
Histamine	0.061 ± 0.006	0.109 ± 0.026	ng/μg total protein	No	*p* = 0.1456
TNF-α	1.310 ± 0.337	0.412 ± 0.046	pg/μg total protein	No	*p* = 0.0575
IL1β	0.054 ± 0.021	0.011 ± 0.004	ng/μg total protein	No	*p* = 0.1151
Catalase	1.135 ± 0.150	0.052 ± 0.007	U/μg total protein	YES	*p* = 0.0019
ROS	1.632 ± 0.153	3.720 ± 0.475	U/μg total protein	YES	*p* = 0.0138
Melatonin	0.970 ± 0.144	0.081 ± 0.007	pg/μg total protein	YES	*p* = 0.0035
Amyloid beta	1.839 ± 0.209	1.334 ± 0.174	ug/μg total protein	No	*p* = 0.1366
p-Tau	1.723 ± 0.181	1.383 ± 0.130	pg/μg total protein	No	p = 0.2014
Hif-1α	2.672 ± 0.216	3.225 ± 0.241	pg/μg total protein	No	*p* = 0.1619
creatinine	0.850 ± 0.023	0.774 ± 0.019	ug/μg total protein	No	*p* = 0.0620
**Muscle**					
leptin	0.096 ± 0.006	0.023 ± 0.001	ng/μg total protein	YES	*p* = 0.0003
Creatine kinase	4.120 ± 0.368	1.100 ± 0.132	ng/μg total protein	YES	*p* = 0.0015
ATP	143.300 ± 11.380	48.960 ± 5.780	ng/μg total protein	YES	*p* = 0.0018
ROS	3.917 ± 0.741	7.427 ± 0.769	U/μg total protein	YES	*p* = 0.0303
Hif-1α	5.708 ± 0.245	8.714 ± 0.522	pg/μg total protein	YES	*p* = 0.0065

Enzymatic- or ELISA (enzyme-linked immunosorbent assay)-based methods were applied to detect the biomarkers brain and in muscle tissues for *lepa* KO zebrafish. The data are expressed as the mean ± SEM and were analyzed by unpaired t-test (*n* control = 3–6; *n lepa* KO = 3–6; significant when *p* < 0.05).

## Supplementary Materials

Supplementary materials can be found at http://www.mdpi.com/1422-0067/19/12/4038/s1. Video S1: Locomotion of wild type (left) and *lepa* KO (right) zebrafish. The three-dimension locomotion trajectories of six fish were recorded and analyzed. This video is played at 10× speed; Video S2: Locomotion of wild type (left) and *lepa* KO (right) zebrafish in novel tank test. The locomotion for 0–1 min (left) and 15–16 min (right) windows were recorded and analyzed by using idTracker software. The upper chamber space is highlighted in yellow color. This video is played at 5× speed; Video S3: Locomotion of wild type (left) and *lepa* KO (right) zebrafish in mirror biting test. The mirror positions are positioned to the right site. The mirror approaching zones are highlighted in yellow color. This video is played at 10× speed; Video S4: Locomotion of wild type (left) and *lepa* KO (right) zebrafish in predator avoidance test. The predator is placed in the left side and zebrafish is placed in the right site and one transparent glass plate is inserted to separate both animals. The predator approaching zones are highlighted in yellow color. This video is played at 10× speed; Video S5: Locomotion of wild type (left) and *lepa* KO (right) zebrafish in social interaction test. The normal zebrafish is placed in the left side and tested KO fish is placed in the right site and one transparent glass plate is inserted to separate both animals. The social interaction approaching zones are highlighted in yellow color. This video is played at 10× speed; Video S6: Locomotion of three wild type (left) and *lepa* KO (right) zebrafish in shoaling test. This video is played at 10× speed.
